# Different Neural Responses to a Moral Valence Decision Task in Unipolar and Bipolar Depression

**DOI:** 10.1155/2013/568617

**Published:** 2013-12-17

**Authors:** Daniele Radaelli, Sara Dallaspezia, Sara Poletti, Enrico Smeraldi, Andrea Falini, Cristina Colombo, Francesco Benedetti

**Affiliations:** ^1^Department of Neuropsychiatric Sciences, Scientific Institute, University Vita-Salute San Raffaele, San Raffaele Turro, Via Stamira d'Ancona 20, Milan, Italy; ^2^Centre of Excellence High Field Magnetic Resonance (C.E.R.M.A.C.), University Vita-Salute San Raffaele, Milan, Italy; ^3^Department of Neuroradiology, Scientific Institute, University Vita-Salute San Raffaele, Milan, Italy

## Abstract

*Objectives*. Patients affected by bipolar disorder (BP) and major depressive disorder (UP) share the susceptibility to experience depression and differ in their susceptibility to mania, but clinical studies suggest that the biological substrates of the two disorders could influence the apparently similar depressive phases. The few brain imaging studies available described different brain metabolic and neural correlates of UP and BP. *Methods*. We studied the BOLD neural response to a moral valence decision task targeting the depressive biases in information processing in 36 subjects (14 BP, 11 UP, and 11 controls). *Results*. Main differences between UP and controls and between UP and BP were detected in left ventrolateral prefrontal cortex (PFC, BA 47). Neural responses of BP patients differed from those of control subjects in multiple brain areas, including anterior cingulate cortex (ACC) and medial PFC, bilateral dorsolateral PFC, temporal cortex and insula, and parietal and occipital cortex. *Conclusions*. Our results are in agreement with hypotheses of dysfunctions in corticolimbic circuitries regulating affects and emotions in mood disorders and suggest that specific abnormalities, particularly in ventrolateral PFC, are not the same in UP and BP depression.

## 1. Objective

Though grouped in the “mood disorders” section of DSM, primary depressive disorder (unipolar depression, UP) and bipolar disorder (BP) show clearly distinctive features, most strikingly because patients share the possibility of experiencing major depression but differ in the susceptibility for mania.

Several findings suggest a biological basis for this difference. Genetic studies confirmed overlapping in the heritability of the two disorders but showed also that approximately 71% of the genetic influence on liability to mania is distinct from the genetic liability to depression [1]. The occurrence of mania seems to be related to alterations in dopaminergic function [2, 3], with CSF homovanillic levels raising before the switch into manic phase [4] and urinary dopamine levels predicting manic mood [5], and it is then hypothesized that the biological mechanisms leading to these changes should be specific of BP.

Treatment options for UP and BP patients are different [6]. A lack of pharmaceutical trials comparing UP and BP prevents definite conclusions, but current opinions suggest different strategies for the treatment of BP and UP depression [7–9], and the clinical evidence is that BP patients experience depressive episodes that are more numerous and less responsive to antidepressant drug treatment [10] with most recent surveys showing that antidepressant drugs that are effective for UP are of little usefulness in BP [11]. Conversely, BP depression is more responsive to chronotherapeutic interventions such as single [12] or repeated [13] sleep deprivation or light therapy [14]. Moreover, the same antidepressant drugs that can be administered to UP patients for years in order to prevent depressive recurrences will precipitate a manic episode in BP patients. Though early comparative studies of the biological distinction between BP and UP depression failed to provide sound evidence [15], these data suggest that the biological differences between the two disorders could not be limited to the occurrence of mania but also influence the apparently similar depressive phases.

Very few studies approached the problem of the UP/BP dichotomy using functional brain imaging techniques. The only blood oxygen level dependent (BOLD) functional magnetic resonance imaging (fMRI) study focused on neural responses to emotional facial expression [16] and confirmed differences between the two conditions. In respect to control subjects BP patients showed increased activations in basal ganglia, hippocampus, ventral striatum, and ventral prefrontal cortex, while UP patients showed a general pattern of decreased response, consistent with hypotheses of “emotional blunting” in UP [17] and limbic-cortical dysregulation [18] with enhanced perceived salience of emotional stimuli in BP.

Dunn et al. [19] correlated (^18^F) fluorodeoxyglucose positron emission tomographic assessment of cerebral metabolism with principal components of the Beck Depression Inventory and found that negative cognitions were associated with decreased frontal metabolism in UP but not in BP patients, while the psychomotor and anhedonic symptoms correlated in both group with higher metabolism in the cingulate cortex and lower metabolism in insula, claustrum, temporal cortex, and basal ganglia, and the overall symptomatology correlated with hypermetabolism in anterior cingulate in both groups. The same research group [20] described an abnormal coupling between regional blood flow and metabolism in anterior cingulate of BP patients, and in large brain regions of UP patients including subgenual, anterior, and posterior cingulate and orbital cortex, these abnormalities are proportional to depression severity in UP, but not BP patients. These findings suggest different brain metabolic correlates of depression and in particular of its cognitive symptomatologic cluster for BP and UP patients.

Cognitive distortions are a regular core symptom of depression and lead to mood-congruent biases in information processing that influence evaluative processes, social judgements, decision-making, attention, and memory. When administered a simple go/no-go task with emotional stimuli, depressed patients show a facilitation of performance when responding to stimuli with a negative emotional tone, showing that congruence between the mood state of the subject and the tone of the stimuli leads to better cognitive performances [21]. BOLD fMRI showed that this task could elicitate different neural responses in depressed patients and healthy controls in medial prefrontal cortex and anterior cingulate, thus suggesting a critical role for these regions in mediating mood-congruent response biases in depression [17]. Previous research by our group in BP depressed patients showed that event-related neural responses to a go/no-go task with morally tuned words in anterior cingulate cortex, dorsolateral prefrontal cortex, insula, and parietal cortex were related to the depressive psychopathological condition, with responders to treatment changing their BOLD responses in a pattern opposite to those of nonresponders [22].

In the present study we compared event-related neural responses to this go/no-go task with morally tuned words in healthy subjects and patients affected by unipolar or bipolar depression.

## 2. Methods

### 2.1. Sample

We studied 14 right-handed patients (5 males, 9 females) with a diagnosis of bipolar disorder type I, depressive episode without psychotic features; 11 right-handed patients (one male, 10 females) with a diagnosis of unipolar depression, and 11 healthy subjects (5 males, 6 females). All Patients were on a depressive phase of the illness.

Control subjects showed an average age of 40.72 years with a standard deviation (sd) of 13.03. Clinical and demographic characteristics of bipolar patients were (mean ± sd) age 46.00 ± 13.66, age at onset 33.36 ± 14.36, number of previous depressive episodes 6.43 ± 7.46, number of previous manic episodes 3.26 ± 2.67, duration of current episode 18.21 ± 14.66 weeks, and severity of depression (21-item Hamilton scale score) 22.29 ± 3.97. Clinical and demographic characteristics of unipolar patients were (mean ± sd) age 46.09 ± 15.20, age at onset 38.81 ± 16.10, number of previous depressive episodes 2.09 ± 1.22, duration of current episode 38.73 ± 36.22 weeks, and severity of depression 25.45 ± 4.46.

Inclusion criteria were absence of mental retardation on Axis II; absence of pregnancy, history of epilepsy, and major medical and neurological disorders; no treatment with long-acting neuroleptic drugs in the last three months before admission; no treatment with neuroleptics in the last month before admission; and absence of a history of drug or alcohol dependency or abuse within the last six months. Depressed patients had an Hamilton Depression Rating Scale (HDRS) score of 18 or higher and no other diagnoses on Axis I.

After a complete description of the study to the subjects a written informed consent was obtained. The study has been reviewed by ethics committee and has therefore been performed in accordance with the ethical standards laid down in the 1964 Declaration of Helsinki.

### 2.2. Image Acquisition

Gradient-echo echo-planar images (EPI) were acquired on a 3.0-Tesla scanner (Gyroscan Intera, Philips, The Netherlands) using a 6-channel SENSE head coil. For each functional run, 200 T2*-weighted axial slices, parallel to the AC-PC plane, were acquired using an EPI pulse sequence (TR = 2200 ms; TE = 35 ms; flip angle = 90°; field of view = 230 mm; number of slices = 18; slice thickness = 4 mm; matrix size = 80 × 80 reconstructed up to 128 × 128 pixels). Two dummy scans before fMRI acquisition allowed obtaining longitudinal magnetization equilibrium. Total time acquisition was 7 minutes and 29 seconds per trial. On the same occasion and using the same magnet 22 Turbo Spin Echo (TSE) T2 axial slices (TR = 3000 ms; TE = 85 ms; flip angle = 90°; turbo factor 15; 5 mm thick, axial slices with a 512 × 512 matrix and a 230 × 230 mm^2^ field of view) were acquired parallel to the AC-PC plane to rule out brain lesions.

### 2.3. Cognitive Activation Paradigm

For each of the four image acquisition sessions, 30 positive and 30 negative for a total of 60 morally tuned adjectives (e.g., brave/vile) were displayed. Each word was presented visually for 1 second. The cognitive activation paradigm was based on a classic go/no-go task. Patients were asked either to push a button for positive targets and ignore negative distractors or the opposite.

The experimental setup was composed of two positive target sessions and two negative target sessions, randomly presented. White colored words were randomly shown on a black screen and presented to the participant through a mirror positioned above the head coil. Emotionally tuned stimuli were interspersed by one, two, or three TRs, following a 4 : 2 : 1 schedule, during which subjects were presented a cross-hair fixation. A 0.1 sec temporal jittering was inserted in order to randomly present every word within the TRs.

### 2.4. Data Processing

Images were computed, overlaid on anatomic images, and analysed using Statistical Parametric Mapping (SPM2. The Wellcome Trust Centre for Neuroimaging, London, England. http://www.fil.ion.ucl.ac.uk/spm/) and Wake Forest PickAtlas [23].

We performed slice timing on all the acquired volumes in order to correct images for time acquisition between first and last slice and realigned the scans to correct for head movement. Data were then normalized to a standard EPI template volume, based on the Montreal Neurological Institute reference brain, and smoothed using a 10 mm full-width at half-maximum isotropic Gaussian kernel. The evoked hemodynamic responses were modelled as a delta function convolved with a hemodynamic response function and its temporal derivative within the context of the general linear model. All events were time locked to the onset of emotionally tuned words.

### 2.5. fMRI Data Analysis

At the individual level, we first compared (*t*-test; threshold *P* < 0.001) both “no-go” and “go” trials to fixation, thereby isolating regions that were engaged by the task during both trial types, and then contrasted “no-go” and “go” images thus creating “double-subtraction” images [(“no-go” > fixation) > (“go” > fixation)] at the single subject level (*t*-test, threshold *P* < 0.001) that were used at the random-effects level.

The resulting four double-subtraction images for each subject (positive/negative) were then entered into a second-level random-effect three-way ANOVA with moral valence of the stimuli and diagnosis as factors. Second-level analyses were thresholded at *P* < 0.01 and limited to gray matter areas. The primary analysis of interest was the two-way interaction, which allowed us to identify the areas were diagnosis and moral valence of the stimuli interacted in influencing the BOLD response to the task.

## 3. Results

Clinical and demographic characteristics of the sample were not significantly different among diagnostic group.

**Figure 1:**
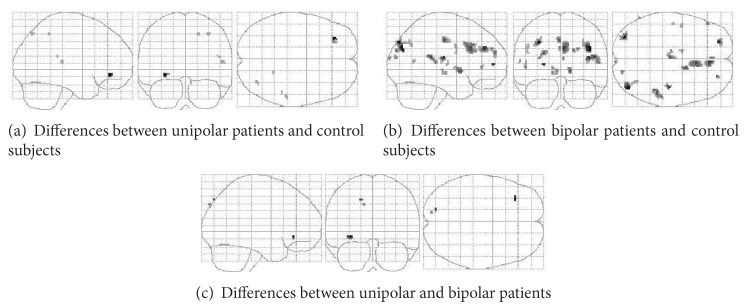
Glass-brain images of gray matter areas where a significant effect of diagnosis and moral valence of the stimuli (negative-positive) was detected.

To test our hypothesis of a different neural correlate of unipolar and bipolar depression to a moral valence decision task, we focused on two-way interaction analyses (with diagnosis and moral valence of the stimuli as independent variables), which allowed us to identify the areas where diagnosis influenced the BOLD response to the moral valence decision task (Table 1 and Figure 1).

**Figure 2:**
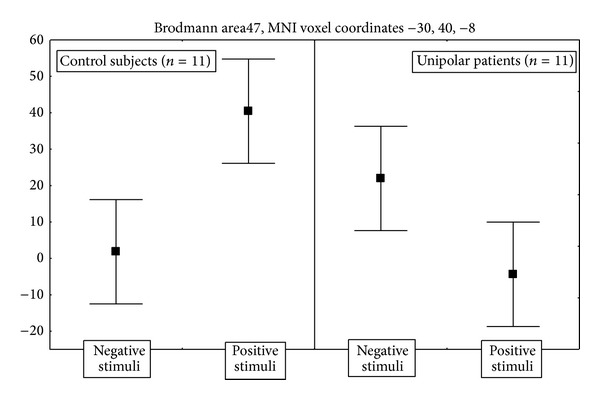
Direction and size effects of the significant interaction of diagnosis (unipolar patients versus control subjects) and moral valence of the stimuli on the event-related BOLD activations in ventrolateral PFC (BA 47, MNI coordinates –30, 40, −8). Points are estimated regression coefficients for the tasks (percent of whole brain mean T2* BOLD signal). Whiskers are standard errors.

In respect to control subjects, UP patients showed significantly different effects of the moral valence of the stimuli in left ventrolateral PFC and right temporal and parietal cortex. Maximal activations were detected in PFC (BA 47), where healthy subjects showed higher activity for positive than for negative stimuli, and UP patients showed the opposite (Figure 2).

**Figure 3:**
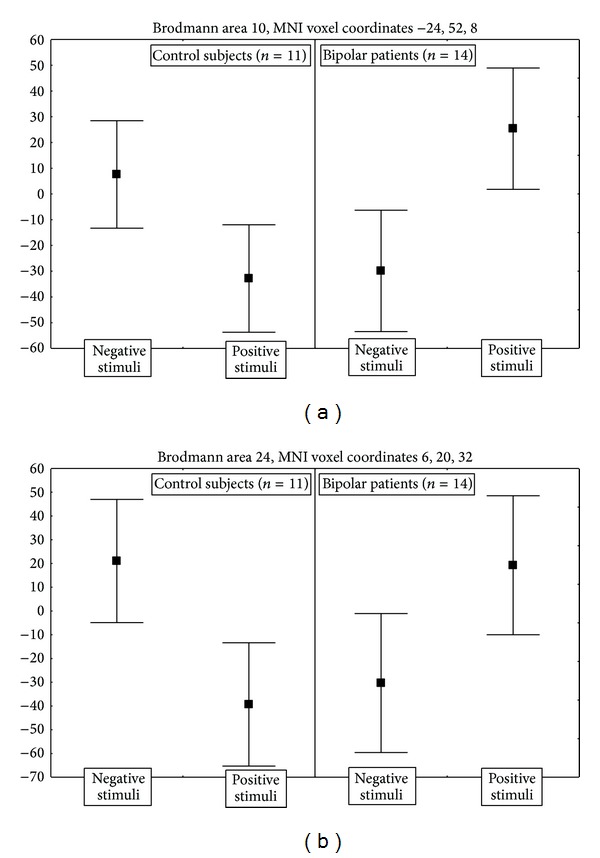
Direction and size effects of the significant interactions of diagnosis (bipolar patients versus control subjects) and moral valence of the stimuli on the event-related BOLD activations in dorsolateral PFC (BA 10), Talairach coordinates 6, 2, 34), and right anterior cingulate cortex (BA 24). Points are estimated regression coefficients for the tasks (percent of whole brain mean T2* BOLD signal) before and after treatment. Whiskers are standard errors.

Differences between BP and control subjects were detected in multiple brain areas, including dorsolateral PFC and a large cluster extending from dorsal and anterior cingulate cortex to medial PFC. In both of these two areas control subjects showed higher responses for negative than for positive stimuli, and bipolar patients showed the opposite (Figure 3).

**Figure 4:**
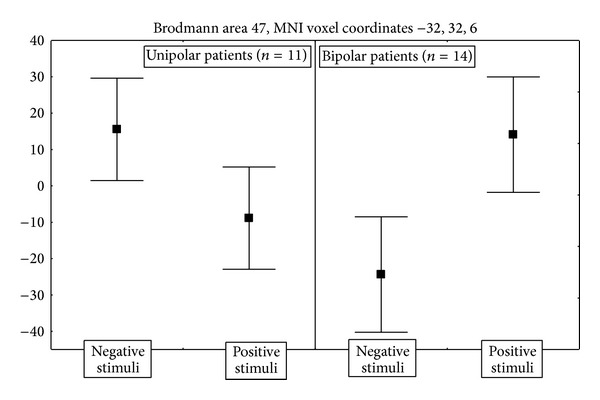
Direction and size effects of the significant interaction of diagnosis (unipolar versus bipolar patients) and moral valence of the stimuli on the event-related BOLD activations in ventrolateral PFC (BA 47, MNI coordinates –32, 32, 6). Points are estimated regression coefficients for the tasks (percent of whole brain mean T2* BOLD signal). Whiskers are standard errors.

Finally, UP and BP patients differed in their pattern of activation to moral stimuli in ventrolateral PFC, where UP patients showed higher activity for negative and BP for positive stimuli (Figure 4).

## 4. Discussion

Despite being all currently affected by a major depressive episode without psychotic features, in respect to healthy subjects, UP and BP patients did not show the same differences in neural correlates to a moral valence decision task.

UP patients showed dissociable response to positive and negative stimuli in respect to both BP and control subjects in left ventrolateral PFC (Figures 2 and 4). Ventrolateral PFC has been implicated in the suppression of sadness [24] and in cognitive tasks involving response inhibition [25] and showed increased activity paralleling transient sadness both in depressed [26] and healthy subjects [27, 28]. It receives projections from visual and somatosensory cortex and shares reciprocal anatomical connections with the amygdala, anterior cingulate cortex, ventral striatum, and hypothalamus, supporting the hypothesis that it participates in integrating experiential stimuli with emotional salience [29, 30], in the identification of emotional stimuli and generation of emotional behaviour [31], and in the linguistic evaluation of emotional stimuli [32].

Concordant PET studies in independent samples [33] comparing UP and control subjects have previously associated familial “pure” depressive disease and increased brain metabolism in an area extending from millimeters from the left of the midline to left medial orbitofrontal and ventrolateral PFC [34]. Studying neural responses to neutral, happy, and sad verbal stimuli with a block design approach, Elliott et al. [17] found that UP patients had enhanced neural responses to mood-congruent stimuli in ventrolateral PFC. Studying the implicit processing of emotional and neutral facial expressions Lawrence et al. [16] found responses to fearful stimuli in the ventrolateral PFC to be different among control, UP, and BP subjects, with BP patients showing higher responses than controls in left BA 47, and UP patients showing higher responses than BP in right BA 47. In BP patients have been reported decreased left ventral PFC volume and glial density have been reported in BP patients [35, 36] who also reported state and trait abnormalities, in neural responses to cognitive activation tasks [37, 38].

In agreement with all these findings of abnormal BA 47 functional neuroanatomy, here we show that neural responses in left ventromedial PFC to a moral decision task distinguish UP patients both from control subjects and from BP patients, thus confirming the relevance of this area and suggesting that its functional abnormalities may contribute to distinguish subtypes of depression. In particular, Drevets et al. [29] hypothesized that increased metabolic activity in ventrolateral PFC during depression could reflect endogenous attempts to attenuate emotional expression or interrupt aversive thoughts and emotions. Our findings of abnormally higher ventrolateral PFC responses to negative stimuli in UP patients (Figure 2) are in agreement with this hypothesis and with findings of ventrolateral PFC activation in response to tasks involving interference resolution [39] and of higher interference from negative than from positive emotional value of verbal stimuli in UP depressed patients [40].

Neural responses of BP patients differed from those of control subjects in multiple brain areas, including large clusters encompassing medial PFC (Figure 3(a)) and anterior cingulate cortex (ACC) (Figure 3(b)). In healthy subjects these areas are widely implicated in the detection of unfavorable outcomes, response errors, response conflict, and decision uncertainty [41] and in resolving emotional conflict [42] and in the present study showed higher activations for negative than for positive stimuli, opposite to BP depressed patients. A wide and consistent literature on PET measures of metabolic activity in perigenual ACC in major depression at baseline and after recovery showed higher metabolic rates at baseline, with a decrease during treatment that was proportional to the clinical amelioration [43, 44]. Structural abnormalities have been detected in right ACC of patients affected by mood disorders, thus allowing a tentative definition of an anatomical endophenotype of bipolar disorder [45]. Neural responses to negative words in ACC increased with response to antidepressant treatment in depressed patients, with their baseline intensity being correlated with response [46], and a previous study by our research group showed that neural responses in ACC to the same cognitive task administered here increased with successful antidepressant treatment in BP patients, with maximal activations being detected at baseline in postrolandic cingulate cortex and after treatment in prerolandic ACC [22].

**Figure 5:**
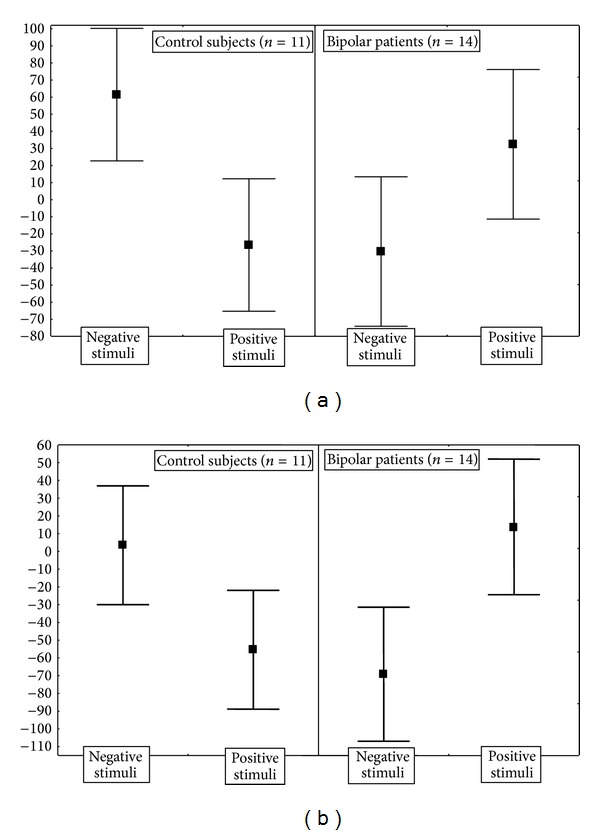
Direction and size effects of the significant interaction of diagnosis (bipolar patients versus control subjects) and moral valence of the stimuli on the event-related BOLD activations in bilateral dorsolateral PFC (BA 10). Points are estimated regression coefficients for the tasks (percent of whole brain mean T2* BOLD signal). Whiskers are standard errors.

In bilateral dorsal-anterolateral PFC (BA 10) BP patients showed higher responses to positive than to negative stimuli, opposite to controls (Figure 5). Sound brain imaging findings showed that dorsolateral PFC is engaged by emotional tasks and plays a major role in the cognitive control of emotions [47] by attenuating the experience of emotion both in the context of voluntary suppression [24] and when emotional distractors interfere with cognitive tasks [48]. Our data suggest that in healthy subjects this “top-down filter” is more engaged by negative than by positive stimuli, while during bipolar depression it is more engaged by positive than by negative stimuli. Consistent with the present findings, in a previous study we showed that successful treatment of bipolar depression reversed this ratio, leading to higher activity in dorsolateral PFC for negative than for positive stimuli, opposite to unsuccessful antidepressant treatment which was unable to produce these changes [22]: that finding can now be interpreted as a normalization of the ratio. In the light of the present case-control comparison, we can then hypothesize that distorted dorsolateral PFC neural responses to the moral valence of verbal stimuli are a correlate of bipolar depression which is normalized with healing and that this abnormal neural reactivity could also contribute to explain the well-known mood-congruent biases in information processing that influence evaluative processes, social judgements, decision-making, attention, memory, and moral reasoning during depressive episodes in bipolar disorder [49].

Other regions where BP patients and control subjects differed included bilateral temporal and right insular cortex and posterior parietal and occipital cortices. Insular cortex activations have been shown to be sensitive to the negative salience of the stimuli [50] with higher responses during transient induction of sadness [24] and in a wide range of negative experiences in healthy and psychiatric populations [51] including the experience of guilt [52].

In conclusion, the present results are in agreement with hypotheses of dysfunctions in corticolimbic circuitries regulating affects and emotions in mood disorders [43, 50, 53] but suggest (I) that specific abnormalities, particularly in ventrolateral PFC, are not the same in UP and BP depression and (II) that functional abnormalities can be detected in the same brain regions with multiple methods (neural response to different activation protocols, PET assessment of brain metabolism, and coupling between metabolism and blood flow), thus suggesting a yet undefined sound structural basis for them.

## Key Points

- Bipolar disorder (BP) major depressive disorder (UP) dichotomy.
- Altered information processing (mood-congruent bias) in mood disorders.
- Neural correlate of UP/BP dichotomy using functional brain imaging techniques.

## Figures and Tables

**Table 1:** Gray matter areas showing a significant interaction of diagnosis and moral valence of the stimuli. Data are shown for the maximal activations in each Brodmann's area (BA): lateralization (L/R), MRIh coordinates (*x*, *y*, *z*) of voxels with higher *Z* values (signal peaks), and level of significance. Glass-brain images of these data are shown in Figure 1.

Comparison	Region	Side	BA	Signal peak	*F*	*Z*	*P*
Unipolar patients versus control subjects	Ventrolateral prefrontal cortex						
	Middle Frontal Gyrus	Left	47	−30 40 −8	13.90	3.35	0.000
	Temporal cortex						
	Superior temporal gyrus	Right	41	54 −28 12	7.81	2.47	0.007
	Insula	Right	13	48 −36 18	7.34	2.38	0.009
	Parietal cortex						
	Superior parietal lobule	Right	7	32 −72 52	7.94	2.49	0.006
	Paracentral lobule	Right	5	20 −48 50	7.90	2.48	0.006
Bipolar patients versus control subjects	Dorsolateral prefrontal cortex						
	Middle frontal gyrus	Left	10	−24 52 8	12.20	3.14	0.001
	Superior frontal gyrus	Right	10	24 50 30	9.50	2.75	0.003
	Medial prefrontal cortex						
	Medial frontal gyrus	Right	9	4 38 30	12.04	3.11	0.001
	Medial frontal gyrus	Left	9	−2 42 32	9.32	2.72	0.003
	Ventrolateral prefrontal cortex						
	Inferior frontal gyrus	Left	45	−50 36 4	9.17	2.70	0.004
	Frontal lobe						
	Precentral gyrus	Left	6	−50 0 30	7.99	2.50	0.006
	Cingulate cortex						
	Anterior	Right	32	6 20 32	11.11	2.99	0.001
	Posterior	Left	24	−2 −18 42	10.59	2.91	0.002
	Temporal cortex						
	Middle temporal gyrus	Right	39	42 −76 28	13.78	3.34	0.000
	Superior temporal gyrus	Right	41	54 −30 10	10.08	2.84	0.002
	Superior temporal gyrus	Left	22	−58 −60 12	8.45	2.58	0.005
	Insula	Right	13	48 −36 18	11.65	3.06	0.001
	Parietal cortex						
	Precuneus	Left	19	−32 −82 40	13.42	3.29	0.000
	Occipital cortex						
	Cuneus	Right	18	4 −96 16	9.92	2.81	0.002
	Middle occipital gyrus	Left	19	−32 −88 16	8.76	2.63	0.004
	Lentiform nucleus						
	Medial globus pallidus	Left		−10 0 2	8.56	2.60	0.005
	Lateral globus pallidus	Right		14 6 −2	10.64	2.92	0.002
	Subthalamic nucleus	Left		−12 –16 −4	9.05	2.68	0.004
Unipolar patients versus bipolar patients	Ventrolateral prefrontal cortex						
	Inferior frontal gyrus	Left	47	−32 32 −6	11.43	3.03	0.001
	Parietal cortex						
	Precuneus	Left	7	−18 −82 46	9.85	2.80	0.003
	Precuneus	Left	19	−12 −88 40	8.57	2.60	0.005
